# Clues to γ-secretase, huntingtin and Hirano body normal function using the model organism *Dictyostelium discoideum*

**DOI:** 10.1186/1423-0127-19-41

**Published:** 2012-04-10

**Authors:** Michael A Myre

**Affiliations:** 1 Molecular Neurogenetics Unit, Center for Human Genetic Research, Department of Neurology, Massachusetts General Hospital, Boston, MA 02114, USA

**Keywords:** *Dictyostelium discoideum*, Model organism, Neurodegeneration, Huntingtin, Presenilin, γ-secretase, Hirano bodies, Neurotransmitter homologues

## Abstract

Many neurodegenerative disorders, although related by their destruction of brain function, display remarkable cellular and/or regional pathogenic specificity likely due to a deregulated functionality of the mutant protein. However, neurodegenerative disease genes, for example *huntingtin *(*HTT*), the *ataxins*, the *presenilins *(*PSEN1/PSEN2*) are not simply localized to neurons but are ubiquitously expressed throughout peripheral tissues; it is therefore paramount to properly understand the earliest precipitating events leading to neuronal pathogenesis to develop effective long-term therapies. This means, in no unequivocal terms, it is crucial to understand the gene's normal function. Unfortunately, many genes are often essential for embryogenesis which precludes their study in whole organisms. This is true for HTT, the β-amyloid precursor protein (APP) and presenilins, responsible for early onset Alzheimer's disease (AD). To better understand neurological disease in humans, many lower and higher eukaryotic models have been established. So the question arises: how reasonable is the use of organisms to study neurological disorders when the model of choice does not contain neurons? Here we will review the surprising, and novel emerging use of the model organism *Dictyostelium discoideum*, a species of soil-living amoeba, as a valuable biomedical tool to study the normal function of neurodegenerative genes. Historically, the evidence on the usefulness of simple organisms to understand the etiology of cellular pathology cannot be denied. But using an organism without a central nervous system to understand diseases of the brain? We will first introduce the life cycle of *Dictyostelium*, the presence of many disease genes in the genome and how it has provided unique opportunities to identify mechanisms of disease involving actin pathologies, mitochondrial disease, human lysosomal and trafficking disorders and host-pathogen interactions. Secondly, I will highlight recent studies on the function of HTT, presenilin γ-secretase and Hirano bodies conducted in *Dictyostelium*. I will then outline the limitations and future directions in using *Dictyostelium *to study disease, and finally conclude that given the evolutionary conservation of genes between *Dictyostelium *and humans and the organisms' genetic tractability, that this system provides a fertile environment for discovering normal gene function related to neurodegeneration and will permit translational studies in higher systems.

## Review

### *Dictyostelium *- emergence of a classic model organism for the study of neurodegenerative disease

**Figure 1:**
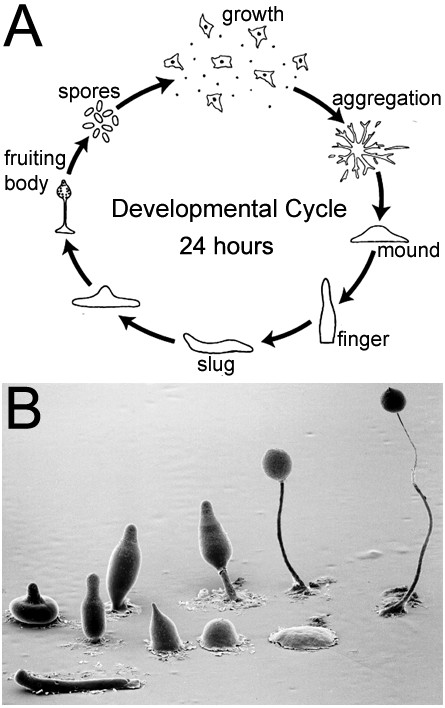
**The life cycle of *Dictyostelium discoideum***. (A) Most of its life exists in its growth phase, as a haploid social amoeba preying upon bacteria in the soil and dividing by mitosis. Once the food source is depleted cells enter into a 24 h multicellular developmental program. During this transition, amoebae aggregate towards secreted cAMP by chemotaxis in the thousands to form a tight mound and then enter a stage where cells begin to differentiate. Cells within the mound remain motile and are directed to differentiate, by secreted morphogens, into either prestalk or prespore cells, culminating to form a fruiting body comprised of a multicellular stalk that supports a ball of encapsulated dormant spores. **(B) **Scanning electron microscopy showing the various structures formed during development. Permissions: CC Creative Commons Attribution - Share Alike 3.0, David Brown & Joan E. Strassmann. SEM courtesy of MJ Grimson & RL Blanton, Biological Sciences Electron Microscopy, Texas Tech University.

*Dictyostelium discoideum *is a species of soil-living amoeba belonging to the Kingdom Amoebozoa and phylum Mycetozoa. *Dictyostelium*, or social amoeba, is a eukaryote that when starved, transitions from individual, self-sustainable professional phagocytes into a highly coordinated developmental program (Figure 1a). During this period of multicellular development, the cells execute a series of morphological changes that proceed in defined stages over a 24 h period to form a *bona fide *multicellular organism. During the earliest developmental stage, cells secrete, and undergo chemotaxis toward cyclic adenosine monophosphate (cAMP) to form aggregation territories. The secretion of cAMP promotes a G protein-coupled receptor signaling cascade that results in the formation of discrete mounds containing as many as 100,000 cells [1,2]. Cells within the mound remain motile and are directed to differentiate into either prestalk or prespore cells, leading to morphogenetic changes yielding a multicellular stalk, supporting a ball of encapsulated dormant spores [3]. The entire process is clearly depicted in Figure 1b by scanning electron microscopy and although the life cycle appears simplistic, the entire process is complex with still many unresolved questions including how do cells secrete cAMP, regulate organism size, and initiate cell-fate choices to name but a few. Nevertheless, it is becoming clearer that *Dictyostelium *possesses signal-transduction pathways that are closely related to metazoans. Yet, with at least one major and exploitable fundamental difference: when animals undergo embryogenesis, they develop through coordinated cell division, morphogenetic movements and differentiation followed by growth of the organism. In contrast, *Dictyostelium *growth precedes development. This simplifies developmental studies and provides a novel route to examine with exceptional cellular clarity biological functions including cytokinesis, endocytosis, secretion, protein trafficking, intra- and extracellular signaling, gene expression, cell-cell communication, adhesion, differentiation and many biochemical aspects of cell motility. With the sequencing of the genome complete [4] we are entering a renaissance in the use of this organism as a biomedical research tool. Bacteria, yeasts and several other invertebrate or vertebrate model systems are known for their contribution to our understanding of basic biology and human disease, but *Dictyostelium*, unfortunately is rarely included among this list. Upon publication of the genome, public availability of RNA seq and DNA microarray data, research using *Dictyostelium *has revealed many common cellular features shared across various phyla, and that many genes encode for proteins more similar to their human counterparts than are those of yeasts (*Saccharomyces cerevisiae*) [4,5]. Yeasts are single, free-living cells that reproduce by budding, whose genome consists of 6,000 genes. Similar to *Dictyostelium*, experiments can be performed over hours, days or weeks whereas in mice these experiments might take years, if at all possible. Moreover, unlike yeasts, the 34 Mb, *Dictyostelium *genome is densely populated with 12, 646 genes and is surprisingly close in size to the 13,676 genes found in the *Drosophila *genome [6]. The fly, unlike *Dictyostelium*, is a multicellular animal with more complex behaviors and nervous system with a lifespan of 2-3 months and generational time of 10 days. Worms (*Caenorhabditis elegans*), also multicellular contains more genes (19,099) than *Dictyostelium *or the fly, a generation time of 3 days and lifespan of 2-3 weeks. Despite their obvious differences, relatively low cost compared to mice, and way of life, importantly, all of these model organisms make proteins that carry out the same core functions as in humans and thus might offer clues to pathogenic processes. Whereas higher eukaryotes often express a number of similar genes with redundant functions, *Dictyostelium*, due to its haploid genome often carries only a single homologous gene making genetic manipulation by targeted gene deletion simple, quick and at a fraction of the cost of higher eukaryotes. A quick scan of the genome reveals many genes that cause neurological disease including, but not limited to adrenoleukodystrophy (*ABCD1*), amyotrophic lateral sclerosis (*SOD1*), Miller-Dieker lissencephaly (*LIS1*), Parkinson's disease (*UCHL1*) and Neuronal ceroid lipofuscinosis (*PPT1, CLN2, CLN3, CLN5*) [4]. A conservative search (threshold value of *e *≤ 10^-40^), detected 64 proteins involved in human diseases [4]. However, searches using advanced programs reveal many orthologous proteins that cause disease (e.g., PSEN1, PSEN2, HTT, Neurofibromatosis 1, LRRK2) such that the number of predicted human disease genes in *Dictyostelium *is greater than previously thought [7-11]. What I hope to convince you of in this short review is the importance of using all available model systems, and posit that, despite its' evolutionary distance, reverse genetics in *Dictyostelium *offers great promise for translational studies aimed at understanding the earliest events leading to neurodegeneration.

### Alzheimer's disease: The amyloid precursor protein and discovery of presenilin-dependent γ-secretase activity

Alzheimer's disease (AD) is the most common cause of dementia, estimated to contribute to about 60 to 70% of cases [12]. AD is a terminal disease that culminates in death. Impaired recent memory usually is an initial symptom of AD, but other cognitive deficits (e.g., changes in attention, problem-solving abilities) may also be present. Amyloid plaques and neurofibrillary tangles, the characteristic pathological hallmarks of AD, are believed to be causative and accrue in the years preceding clinical symptoms. Amyloid plaques are accumulations of insoluble protein aggregates in the extracellular space of the brain and the primary component of plaques is the Aβ peptide, a 38- to 43-amino acid peptide derived from the larger pre-cursor protein APP [13,14]. Mutations in APP [15,16] and two other genes, presenilin-1 (*PSEN1*) [17] and presenilin-2 (*PSEN2*), have been identified that result in autosomal dominant forms of early-onset FAD [18]. The intimate relationship between APP and the presenilins in the production of Aβ is beyond the scope of this review but is detailed elsewhere [19]. The presenilins are homologous, ubiquitously expressed multiple transmembrane domain proteins and many seminal reports provide compelling evidence that presenilins provide the catalytic core of γ-secretase [20-23]. The protein composition of the γ-secretase complexes have been resolved and each consist of four proteins that are all required for proteolytic activity [24-26]: APH-1, PEN2, and nicastrin [25,27]. The normal biological function of the presenilins has been difficult to determine largely due to the fact that it is essential and knockout mice die in utero. To fully understand and appreciate the normal in vivo function of the presenilins they must be studied in the context of organismal models including *Dictyostelium*.

### The identification of presenilins and γ-secretase activity in *Dictyostelium*

**Figure 2:**
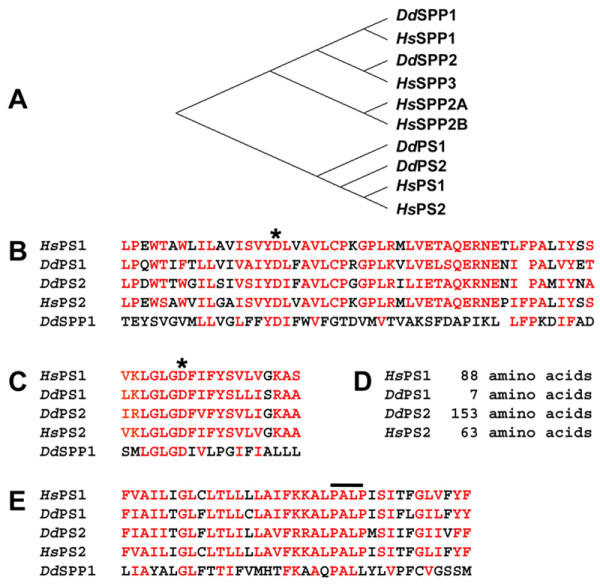
***Dictyostelium *PS proteins align with human presenilins**. (**A**) Phylogenic comparison of amino acid sequences of *Dictyostelium *and the human PS and signal peptidyl proteases (SPP) proteins. The *Dictyostelium *PS proteins cluster closely with human PS. (**B-E**) Sequence alignments of functional domains of PS and SPP proteins. Similar amino acid residues are indicated in red. Residue alignment surrounding the N-terminal enzymatic aspartate (*) is shown. Residue alignment surrounding the C-terminal enzymatic aspartate (*) is shown. Residue alignment within the conserved, *overlined *PALP domain region. Permissions: McMains, V.C. et al. (2010). *Dictyostelium *possesses highly diverged presenilin/γ-secretase that regulates growth and cell-fate specification and can accurately process human APP: a system for functional studies of the presenilin/γ-secretase complex. *Dis. Model. Mech*. 3, pp. 581-594.

A recent development in *Dictyostelium *biomedical research was the identification of homologous genes to *presenilin 1, presenilin 2, nicastrin, aph-1 *and *pen-2*, the core components that constitute the γ-secretase complex [8]. In humans, mutations in the presenilins are intimately involved in the aberrant proteolytic processing of APP in a pathway leading to FAD. How the mutations cause disease, or more importantly the time course or precipitating events that lead to the pathogenesis of AD are not known, and as such, highlight the importance of uncovering the normal function of the γ-secretase complex. In this respect, *Dictyostelium *represents a novel organismal model, to aid in further understanding the normal physiological role of the presenilins and γ-secretase from a conserved and evolutionary point of view in a system that has no Notch or APP equivalent [4]. To study the γ-secretase in *Dictyostelium*, strains were created containing single and/or double gene disruptions for *ps1*^-^, *ps2*^-^, *aph1*^-^, *ncst*^- ^and *ps1*/*ps2*^-^, *aph1*/*ps2*^-^, *ncst*/*ps2*^-^, and *aph*/*ncst*^- ^nulls. It is important to note that mutants of these genes in *Dictyostelium *are viable, unlike the embryonic lethality seen in mammalian systems, and that mutants display a variety of phenotypic defects during specific periods of development. Moreover, phylogenetic sequence analysis reveals that the *Dictyostelium *presenilins are structurally similar to human presenilin rather than the related signal peptidyl proteases (Figure 2A-D), and a majority of mutations in human PSEN causing early-onset FAD [28] are conserved in *Dictyostelium *PSEN proteins [8].

### The presenilin γ-secretase complexes regulate cell differentiation and phagocytosis in *Dictyostelium*

**Figure 3:**
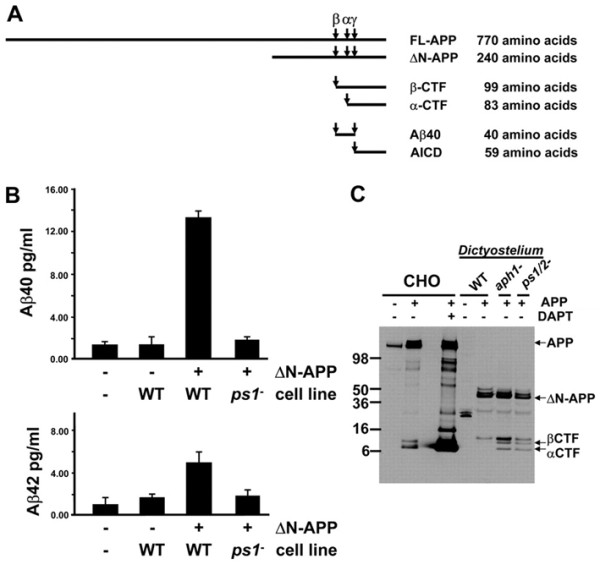
***Dictyostelium *have PS-dependent γ-secretase activity that processes human APP to release Aβ peptides**. (**A**) Schematic showing various APP derived products and their respective sizes. (**B**) ΔN-APP, a truncated human APP expressed in transformed wild type (WT) and ps1-null cells were analyzed for secreted levels of Aβ40 and Aβ42 peptides by quantitative ELISA. Fresh media and media conditioned by native WT cells were used a negative controls. Bars indicate standard errors derived from two independent experiments, each with two replicates. (**C**) Protein samples were collected from native and APP-expressing CHO cells untreated or treated with DAPT, from growing WT *Dictyostelium *or WT, *aph1*-null and *ps1/2*-null *Dictyostelium *that express ΔN-APP. APP expression and processing was determined by immunoblot assay using anti-APP C-terminus antibodies. Permissions: McMains, V.C. et al. (2010). *Dictyostelium *possesses highly diverged presenilin/γ-secretase that regulates growth and cell-fate specification and can accurately process human APP: a system for functional studies of the presenilin/γ-secretase complex. *Dis. Model. Mech*. 3, pp. 581-594.

Cells deficient for *ps1 *did not show post-aggregative developmental defects compared to cells deficient for *ps2 *suggesting that in *Dictyostelium*, PSEN1 γ-secretase complexes may carry out different functional activities than PSEN2 γ-secretase complexes during specific phases of the life cycle. This observation fits well with the suggestion that that there may be several distinct presenilin complexes that have different biological activities [29]. Wild type and *ps1*^- ^cells are able to complete development within 24 h, whereas *ps2*^-^, *aph*1^- ^and *ncst*^- ^mutants not only form fewer fruiting bodies, but predominantly arrest as abnormal intermediate structures within the same time frame [8]. The authors also show that in *Dictyostelium *γ-secretase complexes function to regulate cell differentiation as measured by cell-type specific gene expression and firmly establish that presenilin γ-secretase activity is required in a cell-autonomous pathway that determines cell fate during development [8]. Once again, these in vivo observations support a large body of suggestive evidence for presenilin γ-secretase playing an essential role in cellular and neuronal differentiation during neural development and adult neurogenesis in mouse and zebrafish models [30,31]. Cells deficient for γ-secretase also display a reduced ability for phagocytosis. Quantification of phagocytic rates for the various mutant strains revealed that cells lacking PSEN1 had highly reduced rates of phagocytosis, but this was not the case for *ps2*^- ^cells. The described phagocytosis defect supports observations that mammalian presenilins are present in lysosomal membranes, but also strengthens the argument that distinct presenilin complexes exist and have different biological activities. Further to this, when wild type amoeba were made to express a variant of human APP, the cells were clearly able to process APP in a manner identical to mammalian cells resulting in the differential production of C-terminal fragments and Aβ40/Aβ42, the toxic fragment believed to be the causative agent in AD (Figure 3).

This work demonstrates the evolutionary conservation of this key regulatory enzyme. The most obvious value stemming from this work for the study of presenilin function are the possibilities for elucidating presenilin-independent function(s) as wild-type presenilins are required for proper protein degradation through the autophagosome-lysosome system thereby implicating a role for these proteins in autophagy [32]; secondly, the exciting prospects surrounding the finding that proteolytic processing of APP occurs in *Dictyostelium *exactly the same way as has been shown animal cells. This finding is important not only because it provides an excellent system in which to test therapeutic agents that target Aβ production in vivo, but it is remarkable that this enzyme, given the evolutionary distance between *Dictyostelium *and humans, is capable of cleaving a non-endogenous substrate to produce C-terminal fragments and ratios of secreted Aβ40/Aβ42 identical to mammalian systems. Knowledge gained about human diseases including cancers from the use of yeast cannot be denied, and with *Dictyostelium*, we also have an equally genetically tractable organism that not only contains genes uncannily similar to humans, but as a single cell behaves much like an animal cell and further offers the ability to study gene function under conditions of multicellularity all in the same model. Even with the evolutionary distance between humans and *Dictyostelium *one might expect genes to play different roles, but with regard to amyloidogenic processing of APP, this does not appear to be the case. It should be mentioned that a survey of the *Dictyostelium *genome does not readily identify or predict the presence of either the known α-secretase or β-secretase [4] and suggests the presence of yet to be identified first-cleavage sheddases either unique to *Dictyostelium *or potentially conserved across metazoan species. Moreover, this model offers a biochemical clarity not offered by any other system to date to study γ-secretase function directly, free from the multiple secondary effects that can arise from aberrant proteolytic processing of more than 60 identified substrates [33]. This study provides strong in vivo evidence for presenilin function in regulating phagocytosis, only previously suspected in animal cell culture [34]. Lastly, with the availability of *Dictyostelium *strains carrying multiple loss-of-function mutations to the components of the γ-secretase, this model system allows for the rapid systematic analysis of how specific mutations to human presenilins might result in gain-of-function or loss-of-function effects that can be readily assessed in higher models. Further studies will be required to determine whether the mammalian *PSEN *gene can rescue *Dictyostelium ps1*-null phenotypes and proteolytic processing of human APP.

### Higher organism and cellular models of Huntington's disease

Huntington's disease (HD) is a fatal neurodegenerative disease [35] also known as Huntington's chorea, that affects muscle coordination from preferential loss of efferent medium spiny neurons in the striatum of the basal ganglia, leads to cognitive decline, dementia and eventually death [36-38]. Genetic evidence suggests the disease is caused by an unstable CAG trinucleotide repeat within the coding region of the *htt *gene leading to expansion (> 35) of glutamine (Q) residues near the amino-terminus of the protein [35]. Mutant HTT confers an unknown gain-of-function property to the protein, but might also impair normal protein function [39]. Huntingtin is a large (350 kDa) alpha-solenoid HEAT (*h*untingtin, *e*longation factor 3, the *A *subunit of protein phosphatase 2A, and *T*OR1) repeat protein [40] with little resemblance to any known protein. The normal function of HTT, the nature of the polyQ mutation which is expressed in both neuronal and non-neuronal cells and how it contributes to pathogenesis still remains unclear [41]. Although genetically precise mouse models for HD have contributed significantly towards understanding HTT normal function, these mice are expensive to create, to maintain and therefore difficult to recapitulate results. Thus, defining the disease-producing 'gain-of-function' - either a polyglutamine-length dependent increase or deregulation of a normal huntingtin activity or the introduction of a novel polyglutamine-length dependent activity, will require an understanding of the protein's normal function(s).

A wide range of HD animal models are currently available and have been used to investigate pathological pathways, molecular targets, and potential therapeutics. Invertebrates such as *Drosophila melanogaster*, non-mammalian species (e.g., *Danio rerio*) and mammals, including mice, rats, sheep and non-human primates, have been genetically engineered to model the HD mutation. Although these systems are extremely important tools to understand HD, a large majority of studies focus upon the late end-stage disease symptoms and thus offer minimal insight into early manifestation including HTT normal function. The existence of HD cell lines has been, to a finite degree, useful for the dissection of huntingtin function and assessment of potential therapeutic compounds [42,43]. Further, in vitro cell lines often do not show overt defects suggesting they are unable to reproduce the pathophysiological mechanisms induced by the mutant gene.

Many established model systems are based upon an amino-terminal fragment that results in perturbation of many cellular processes but these remove the polyglutamine tract from its normal protein context. Therefore, one cannot understate that strategies are badly needed to reveal full length huntingtin-dependent biochemical processes that contribute to HD pathology. Because of the evolutionary conservation of huntingtin, lower organism systems offer an attractive and affordable route that has yet to be exploited to understand huntingtin function.

### The identification of *Dictyostelium *huntingtin

The *Dictyostelium *genome was screened and found to contain a single gene with sequence homology to human huntingtin. Bioinformatic and phylogenetic analysis of the primary amino acid sequence placed the protein firmly within the huntingtin family, including size, and the presence of numerous HEAT and HEAT-like repeats. *Dictyostelium *mutants' deficient for HTT cells are viable, unlike the embryonic lethality seen in higher eukaryotes, but are fragile in that they display a number of subtle phenotypes suggesting that HTT is involved in a number of cellular processes [9]. We show that huntingtin deficiency impacts upon osmoregulation, cation homeostasis, cell motility, cell shape, chemotactic cAMP relay, homotypic cell-cell adhesion, cell fate determination and cytokinesis in *Dictyostelium *[9,44]. Importantly, these phenotypic deficits are greatly dependent upon the environment in which the cells are placed which suggests that HTT aids in translating extracellular signals into cellular processes. These findings support the large body of evidence that suggest in metazoa, huntingtin is a multifunctional protein with roles in embryogenesis, cell fate, cytoskeleton, apoptosis, vesicular and mitochondrial transport, iron homeostasis, autophagy, energy metabolism and transcriptional regulation [39,41,45,46]. A major issue of concern is which, if any, of these functions directly involve HTT or are secondary effects to HTT deficiency or mutant HTT activity. The power behind the *Dictyostelium *system is that it allows for the identification of in vivo HTT functions at the level of both single cells and a multi-cellular environment all in the same organism with a biochemical and cellular clarity to identify functions that directly depend on HTT.

### *Dictyostelium *cells deficient for huntingtin display cell shape and cytoskeletal defects

When *htt*^- ^cells are placed under low ionic strength phosphate buffer, the cells elicit a round phenotype without membrane extensions, a reduction of F-actin at the cortex of the cell and under hypoosmotic conditions rapidly swell and undergo complete lysis within 5-6 h [9]. This fits well with reports that mutant HTT in cells from HD patients or when normal huntingtin levels are reduced, cells display defective actin-remodeling under conditions of stress [47] and consistent with mammalian studies that huntingtin regulates neurological processes including actin-rich dendritic spine formation and membrane branching [48,49]. In vitro binding of the HTT N-terminus to F-actin has been reported [50] and may indicate a normal, perhaps transient, function for HTT in the regulation of the actin cytoskeleton. *Dictyostelium *provides a valuable tool to uncover mechanisms by which HTT regulates actin-cytoskeleton responses.

### Huntingtin modulates chemotaxis in *Dictyostelium*

The failure of *htt*^- ^cells to osmoregulate and sensitivity to extreme hypoosmotic shock secondarily affects intracellular ion homeostasis. As a consequence, unless specific cations (Ca^2+ ^or Mg^2+^) are provided exogenously *htt*^- ^cells appear unable to initiate cAMP-induced Ca^2+^-transients that may act in a feedback loop to positively reinforce cAMP relay which impairs chemotaxis during *Dictyostelium *development. Although the a role for HTT in chemotaxis has not been established in mammalian systems, a consensus is gradually emerging that the dyshomeostasis of Ca^2+ ^is an important factor in the linkage of the HTT mutation to the onset and progression of the disease [51] and together suggest abnormal Ca^2+ ^signaling and other Ca^2+ ^signaling proteins should be explored further as an evolutionary conserved role for HTT.

### Huntingtin regulates cell fate during development in *Dictyostelium*

**Figure 4:**
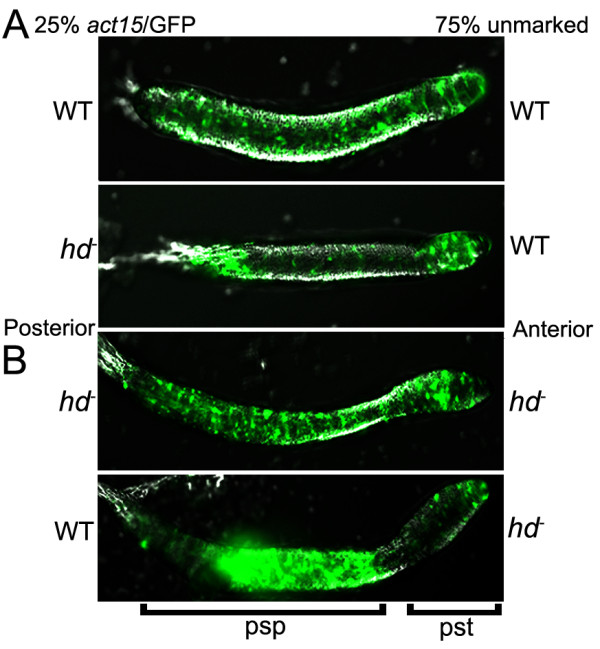
**Huntingtin regulates prespore/spore differentiation cell-autonomously**. (A) GFP was expressed in *htt*^- ^cells and their position in chimeras with unlabelled wild type cells was monitored. GFP:*htt*^- ^cells fail to populate the prespore domain. (B) Reverse experiment in which GFP: WT cells in chimeras with unlabelled *htt*^- ^cells occupy the prespore domain. The prestalk (pst) and prespore (psp) domains are *underlined*. Cell types within each region are shown and slugs are arranged with the front facing towards the right. Permission: Myre M.A. et al. (2009). Deficiency of huntingtin has pleiotropic effects in the social amoeba *Dictyostelium discoideum. PLoS Genetics*, 7(4), pp. e1002052.

It was shown that HTT modulates cell fate in *Dictyostelium *(Figure 4). Using chimeras where cells are marked with GFP, *htt*^- ^cells in the presence of wild type cells fail to populate the prespore region of the slug, and as a consequence do not become spores [9]. Cell fate defects in huntingtin deficient cells have also been observed in vertebrates. In zebrafish, reduced expression of huntingtin differentially targets development of telencephalic neurons compared to mid- and hind-brain [52]. In mouse chimeras, *Hdh*-/- cells preferentially colonize the hypothalamus, midbrain, and hindbrain relative to the telencephalon and the thalamus during early development [53]. Thus, like these latter neuronal populations, *Dictyostelium *cells require huntingtin for the proper development of viable spores. Not surprisingly, the data thus far do not provide a simple definition for a single normal function for huntingtin, but, as huntingtin deficiency in *Dictyostelium *produces pleiotropic effects, the findings are consistent with the consensus from mammalian studies that huntingtin is a multifunctional protein that impacts upon many biochemical processes. Exciting new avenues of research have emerged in delineating which of these functions are conserved in mammals. Ultimately, determining to what degree these functions are altered by expansion of the polyglutamine tract in human huntingtin will provide much needed insights into the mechanism by which mutant huntingtin triggers HD pathogenesis.

### Insight into Hirano bodies - *Dictyostelium discoideum*

Hirano bodies, described about 40 years ago, are bright eosinophilic intracytoplasmic inclusions/protein aggregates with crystalloid fine rod structures that occur preferentially in the neuronal processes of individuals with neurodegenerative diseases including Alzheimer's disease, Creutzfeldt-Jacob disease, amyotrophic lateral sclerosis, parkinsonism-dementia and Pick's disease [54,55]. Hirano bodies are largely considered a pathological hallmark of postmortem brain samples from patients suffering from neurodegenerative disorders representing "tombstones" of neuronal cell death. Hirano bodies have been reported in glial cells, in peripheral nerve axons and in extraocular muscles of eyes [56] suggesting they are more than products of cell death. It should be noted that Hirano bodies, as a function of age, appear in individuals without neurodegeneration [57]. Hirano bodies are complex inclusions comprised of many proteins including actin and actin-associated proteins (cofillin and alpha-actinin), tau, a C-terminal fragment of APP; microtubule associated bundling proteins, and neurofilaments [55]. Moreover, they have been described in various experimental animals. Understanding the underlying pathological molecular mechanism responsible for their formation has been precluded by the lack of either an in vitro or in vivo experimental model system.

Research using *Dictyostelium *revealed that formation of Hirano bodies is a pathological event [58]. The expression in *Dictyostelium *of the CT fragment (amino acids 124-295) of the 34 kDa protein, the 34 kDa protein is one of 11 actin crosslinking proteins present in *Dictyostelium*, a protein that exhibits activated actin binding and calcium-insensitive actin filament cross-linking activity induced cells to form ellipsoidal paracrystalline inclusions that contain ordered assemblies of F-actin, CT-myc, myosin II, cofilin and alpha-actinin that remarkably resembled the structure of Hirano bodies. They extended their findings to mammalian murine cells and discovered that F-actin rearrangements caused by a reduction in stress fibers accumulate as numerous punctate foci and large aggregates: Hirano bodies [58]. So the failure to regulate either the activity and/or affinity of an actin cross-linking protein provides a signal that forms Hirano bodies. Whereas wild type cells complete development in 24 h, CT-myc cells contained significantly higher amounts of F-actin, formed fruiting bodies with delayed developmental timing by ~6 h. The accumulation of F-actin was also shown to occur in murine cells expressing CT-myc. This report established for the first time that Hirano bodies are not restricted to mammalian cells or nerve cells occur because of aberrant function of the actin cytoskeleton and that Hirano bodies can exert defects on normal cell function. Because of using *Dictyostelium*, Hirano bodies and induction of F-actin rearrangements in mammalian cell cultures by expression of the CT 34-kDa actin-bundling protein was found to occur in HEK 293, HeLa, Cos7 cells, neuroblastoma and astrocytic cells, and in primary neurons [59].

## Conclusion

Several *Dictyostelium *genes are homologous or retain a significant amount of structural conservation to human genes making the organism a useful biomedical model system. With the entire genome sequenced and publicly available in a model organism database called dictyBase, it allows molecular and cellular biologists to examine with more clarity the complex multifunctional aspects of gene function. However, one limitation of using unicellular organisms (e.g., yeast) as models for assessing the function of genes involved in disease is that pathologies which affect specific organs cannot be as readily assessed as it might be in the relevant organism. This limitation may also apply to genes that are present in unicellular organisms but are required in a more stringent manner in specialized tissues, or expressed as a variety of different isoforms in specific cell types. However, this limitation has not precluded the use of lower organisms to understand cell division and cancers in many different organs. Although *Dictyostelium *develops into *bona fide *multicellular organism, it does present a limitation in the number of cell types that can be analyzed and therefore discoveries made using this organism must be rigorously assessed in the relevant mammalian cell type. Coupled with translational experiments and validation in higher eukaryotes, research using *Dictyostelium *will expedite and contribute to our understanding of human neurological diseases. Individual cell behavior accounts for the many phases of health and pathogenic mechanisms that initiate disease. This has been elegantly portrayed in *Dictyostelium *with respect to the precise production of Aβ from the heterologous expression of human APP. Chemotaxis is a critical cellular function with implications in embryogenesis. *Dictyostelium *cells deficient for HTT show highly defective chemotactic behavior coupled with cytoskeletal/membrane deficits under conditions of low ionic conditions that may reveal conserved roles for this protein in axon guidance, neuritic extensions and embryogenesis. Future endeavors that increases the amount of translational research conducted in higher organisms or neuronal cell models will be beneficial for understanding both *Dictyostelium *cellular physiology and, more importantly, the cellular mechanisms of human disease. I wish to posit that multiple model systems can and should be employed in the cross-genomic analysis of human neurodegenerative disease genes to address multiple basic eukaryotic cellular functions (e.g., *Dictyostelium*), to their assembly into various types of more complex molecular pathways (e.g., flies and worms), and then validated and assessed in models of human neurodegenerative disease (e.g., mice).

## Abbreviations

HTT: Huntingin; PSEN1: Presenilin 1; PSEN2: Presenilin 2; APP: Amyloid precursor protein; AD: Alzheimer's disease; HD: Huntington's disease; cAMP: Cyclic adenosine monophosphate; FAD: Familial Alzheimer's disease; HEAT: Huntingtin: elongation factor 3: the A subunit of protein phosphatase 2A: and TOR1.

## Competing interests

The author declares that they have no competing interests.

## Authors' contributions

MAM contributed to the conception, design and drafting of this manuscript.

## References

[B1] Parent CA Devreotes P Molecular genetics of signal transduction in *Dictyostelium* Annu Rev Biochem 1996 65 411 440 10.1146/annurev.bi.65.070196.002211 8811185

[B2] Soderbom F Loomis B Cell-cell signaling during *Dictyostelium *development Trends Microbiol 1998 6 10 402 406 10.1016/S0966-842X(98)01348-1 9807784

[B3] Williams JG Duffy KT Lane DP McRobbie SJ Harwood AJ Traynor D Kay RR Jermyn KA Origins of the prestalk-prespore pattern in *Dictyostelium *development Cell 1989 59 6 1157 1163 10.1016/0092-8674(89)90771-X 2513127

[B4] Eichinger L Pachebat JA Glöckner G Rajandream MA Sucgang R Berriman M Song J Olsen R Szafranski K Xu Q Tunggal B Kummerfeld S The genome of the social amoeba *Dictyostelium discoideum* Nature 2005 435 43 57 10.1038/nature03481 15875012 PMC1352341

[B5] Parikh A Miranda ER Katoh-Kurasawa M Fuller D Rot G Zagar L Curk T Sucgang R Chen R Zupan B Loomis WF Kuspa A Shaulsky G Conserved developmental transcriptomes in evolutionarily divergent species Genome Biol 2010 11 3 R35 10.1186/gb-2010-11-3-r35 20236529 PMC2864575

[B6] Adams MD Celniker SE Holt RA Evans CA Gocayne JD Amanatides PG Scherer SE Li PW Hoskins RA Galle RF George RA Lewis SE The genome sequence of *Drosophila melanogaster* Science 2000 287 5461 2185 2195 10.1126/science.287.5461.2185 10731132

[B7] Bosgraaf L Van Haastert PJ Roc, a Ras/GTPase domain in complex proteins Biochim Biophys Acta 2003 1643 5 10 10.1016/j.bbamcr.2003.08.008 14654223

[B8] McMains VC Myre MA Kreel L Kimmel AR *Dictyostelium *possesses highly diverged presenilin/gamma-secretase that regulates growth and cell-fate specification and can accurately process human A: a system for functional studies of the presenilin/γ-secretase complex Dis Models Mech 2010 3 9-10 581 594 10.1242/dmm.004457 PMC2931536 20699477

[B9] Myre MA Lumsden AL Thompson MN Wasco W Macdonald ME Gusella JF Deficiency of huntingtin has pleiotropic effects in the social amoeba *Dictyostelium discoideum* PLoS Genet 2011 7 4 e1002052 10.1371/journal.pgen.1002052 21552328 PMC3084204

[B10] Wang Y Steimle PA Ren Y Ross CA Robinson DN Egelhoff TT Sesaki H Iijima: Dictyostelium huntingtin controls chemotaxis and cytokinesis through the regulation of myosin II phosphorylation M Mol Biol Cell 2011 22 13 2270 2281 10.1091/mbc.E10-11-0926 PMC3128529 21562226

[B11] Zhang S Charest PG Firtel RA Spatiotemporal regulation of Ras activity provides directional sensing Curr Biol 2008 18 20 1587 1593 10.1016/j.cub.2008.08.069 18948008 PMC2590931

[B12] Barker WW Luis CA Kashuba A Luis M Harwood DG Loewenstein D Waters C Jimison P Shepherd E Sevush S Graff-Radford N Newland D Relative frequencies of Alzheimer disease, Lewy body, vascular and frontotemporal dementia, and hippocampal sclerosis in the State of Florida Brain Bank Alzheimer Dis Assoc Disord 2002 16 4 203 212 10.1097/00002093-200210000-00001 12468894

[B13] Golde TE Eckman CB Younkin SG Biochemical detection of Abeta isoforms: implications for pathogenesis, diagnosis, and treatment of Alzheimer's disease Biochim Biophys Acta 2000 1502 1 172 187 10899442 10.1016/s0925-4439(00)00043-0

[B14] Hardy J Selkoe DJ The amyloid hypothesis of Alzheimer's disease: progress and problems on the road to therapeutics Science 2002 297 5580 353 356 10.1126/science.1072994 12130773

[B15] Goate A Chartier-Harlin MC Mullan M Brown J Crawford F Fidani L Giuffra L Haynes A Irving N James L Segregation of a missense mutation in the amyloid precursor protein gene with familial Alzheimer's disease Nature 1991 349 704 706 10.1038/349704a0 1671712

[B16] Levy E Carman MD Fernandez-Madrid IJ Power MD Lieberburg I van Duinen SG Bots GT Luyendijk W Frangione B Mutation of the Alzheimer's disease amyloid gene in hereditary cerebral hemorrhage, Dutch type Science 1995 248 1124 1126 2111584 10.1126/science.2111584

[B17] Levy-Lahad E Wasco W Poorkaj P Romano DM Oshima J Pettingell WH Yu CE Jondro PD Schmidt SD Wang K Crowley AC Ying-Hui F Guenette SY Galas D Nemens E Wijsman EM Bird TD Schellenberg GD Tanzi RE Candidate gene for the chromosome 1 familial Alzheimer's disease locus Science 1995 269 973 977 10.1126/science.7638622 7638622

[B18] Sherrington R Rogaev EI Liang Y Rogaeva EA Levesque G Ikeda M Chi H Lin C Li G Holman K TSuda T Mar L Cloning of a gene bearing missense mutations in early-onset familial Alzheimer's disease Nature 1995 375 754 760 10.1038/375754a0 7596406

[B19] Zhang YW Thompson R Zhang H Xu H APP processing in Alzheimer's disease Mol Brain 2011 4 3 10.1186/1756-6606-4-3 21214928 PMC3022812

[B20] De Strooper B Saftig P Craessaerts K Vanderstichele H Guhde G Annaert W Von Figura K Van Leuven F Deficiency of presenilin-1 inhibits the normal cleavage of amyloid precursor protein Nature 1998 391 387 390 10.1038/34910 9450754

[B21] De Strooper B Annaert W Cupers P Saftig P Craessaerts K Mumm JS Schroeter EH Schrijvers V Wolfe MS Ray WJ Goate A Kopan R A presenilin-1-dependent gamma-secretase-like protease mediates release of Notch intracellular domain Nature 1999 398 518 522 10.1038/19083 10206645

[B22] Struhl G Greenwald I Presenilin is required for activity and nuclear access of Notch in *Drosophila* Nature 1999 398 6727 522 525 10.1038/19091 10206646

[B23] Wolfe MS Xia W Ostaszewski BL Diehl TS Kimberly WT Selkoe DJ Two transmembrane aspartates in presenilin-1 required for presenilin endoproteolysis and gamma-secretase activity Nature 1999 398 513 517 10.1038/19077 10206644

[B24] De Strooper B Aph-1, Pen-2, and Nicastrin with Presenilin generate an active gamma-Secretase complex Neuron 2003 38 1 9 12 10.1016/S0896-6273(03)00205-8 12691659

[B25] Edbauer D Winkler E Regula JT Pesold B Steiner H Haass C Reconstitution of gamma-secretase activity Nat Cell Biol 2003 5 486 488 10.1038/ncb960 12679784

[B26] Kimberly WT LaVoie MJ Ostaszewski BL Ye W Wolfe MS Selkoe DJ Gamma-secretase is a membrane protein complex comprised of presenilin, nicastrin, Aph-1, and Pen-2 Proc Natl Acad Sci USA 2003 100 11 6382 6387 10.1073/pnas.1037392100 12740439 PMC164455

[B27] Yu G Nishimura M Arawaka S Levitan D Zhang L Tandon A Song YQ Rogaeva E Chen F Kawarai T Supala A Levesque L Nicastrin modulates presenilin-mediated notch/glp-1 signal transduction and Aβ processing Nature 2000 407 6800 48 54 10.1038/35024009 10993067

[B28] Kim SD Kim J Sequence analyses of presenilin mutations linked to familial Alzheimer's disease Cell Stress Chaperones 2008 13 401 412 10.1007/s12192-008-0046-0 18491041 PMC2673935

[B29] Gu Y Sanjo N Chen F Hasegawa H Petit A Ruan X Li W Shier C Kawarai T Schmitt-Ulms G Westaway D St. George-Hyslop P Fraser PE The presenilin proteins are components of multiple membrane-bound complexes that have different biological activities J Biol Chem 2004 279 30 31329 31336 10.1074/jbc.M401548200 15123598

[B30] Baumeister R The physiological role of presenilins in cellular differentiation: lessons from model organisms Eur Arch Psychiatry Clin Neurosci 1999 249 6 280 287 10.1007/s004060050100 10653283

[B31] van Tijn P Kamphuis W Marlatt MW Hol EM Lucassen PJ Presenilin mouse and zebrafish models for dementia: focus on neurogenesis Prog Neurobiol 2011 93 2 149 164 10.1016/j.pneurobio.2010.10.008 21056616

[B32] Neely KM Green KN LaFerla FM Presenilin is necessary for efficient proteolysis through the autophagy-lysosome system in a γ-secretase-independent manner J Neurosci 2011 31 8 2781 2791 10.1523/JNEUROSCI.5156-10.2010 21414900 PMC3064964

[B33] McCarthy JV Twomey C Wujek P Presenilin-dependent regulated intramembrane proteolysis and γ-secretase activity Cell Mol Life Sci 2009 66 9 1534 1555 10.1007/s00018-009-8435-9 19189053 PMC11131470

[B34] Jutras I Laplante A Boulais J Brunet S Thinakaran G Desjardins M γ-secretase is a functional component of phagosomes J Biol Chem 2005 280 43 36310 36317 10.1074/jbc.M504069200 16103123

[B35] The Huntington's Disease Collaborative Research Group A novel gene containing a trinucleotide repeat that is expanded and unstable on Huntington's Disease chromosomes Cell 1993 72 971 983 10.1016/0092-8674(93)90585-E 8458085

[B36] Reiner A Albin RL Anderson KD D'Amato CJ Penney JB Young AB Differential loss of striatal projection neurons in Huntington disease Proc Natl Acad Sci USA 1998 85 15 5733 5737 2456581 10.1073/pnas.85.15.5733 PMC281835

[B37] Rosas HD Koroshetz WJ Chen YI Skeuse C Vangel M Cudkowicz ME Caplan K Marek K Seidman LJ Makris N Jenkins BG Goldstein JM Evidence for more widespread cerebral pathology in early HD: an MRI-based morphometric analysis Neurology 2003 60 10 1615 1620 12771251 10.1212/01.wnl.0000065888.88988.6e

[B38] Rosas HD Hevelone ND Zaleta AK Greve DN Salat DH Fischl B Regional cortical thinning in preclinical Huntington disease and its relationship to cognition Neurology 2005 65 5 745 747 10.1212/01.wnl.0000174432.87383.87 16157910

[B39] Cattaneo E Zuccato C Tartari M Normal huntingtin function: An alternative approach to Huntington's disease Nat Rev Neurosci 2005 6 12 919 930 16288298 10.1038/nrn1806

[B40] Andrade MA Bork P HEAT repeats in the huntingtin protein Nat Genet 1995 11 2 115 116 10.1038/ng1095-115 7550332

[B41] Zuccato C Valenza M Cattaneo E Molecular mechanisms and potential therapeutical targets in Huntington's disease Physiol Rev 2010 90 3 905 981 10.1152/physrev.00041.2009 20664076

[B42] Rigamonti D Bolognini D Mutti C Zuccato C Tartari M Sola F Valenza M Kazantsev AG Cattaneo E Loss of huntingtin function complemented by small molecules acting as repressor element 1/neuron restrictive silencer element silencer modulators J Biol Chem 2007 282 34 24554 24562 10.1074/jbc.M609885200 17565993

[B43] Varma H Voisine C DeMarco CT Cattaneo E Lo DC Hart AC Stockwell BR Selective inhibitors of death in mutant huntingtin cells Nat Chem Biol 2007 3 2 99 100 10.1038/nchembio852 17195849

[B44] Wang Y Steimle PA Ren Y Ross CA Robinson DN Egelhoff TT Sesaki H Iijima Dictyostelium huntingtin controls chemotaxis and cytokinesis through the regulation of myosin II phosphorylation M Mol Biol Cell 2011 22 13 2270 2281 10.1091/mbc.E10-11-0926 PMC3128529 21562226

[B45] Lumsden AL Henshall TL Dayan S Lardelli MT Richards RI Huntingtin-deficient zebrafish exhibit defects in iron utilization and development Hum Mol Genet 2007 16 16 1905 1920 10.1093/hmg/ddm138 17567778

[B46] Imarisio S Carmichael J Korolchuk V Chen CW Saiki S Rose C Krishna G Davies JE Ttofi E Underwood BR Rubinsztein DC Huntington's disease: from pathology and genetics to potential therapies Biochem J 2008 412 2 191 209 10.1042/BJ20071619 18466116

[B47] Munsie L Caron N Atwal RS Marsden I Wild EJ Bamburg JR Tabrizi SJ Truant R Mutant huntingtin causes defective actin remodeling during stress: defining a new role for transglutaminase 2 in neurodegenerative disease Hum Mol Genet 2011 20 10 1937 1951 10.1093/hmg/ddr075 21355047 PMC3080606

[B48] Dent EW Merriam EB Hu X The dynamic cytoskeleton: backbone of dendritic spine plasticity Curr Opin Neurobiol 2011 21 1 175 181 10.1016/j.conb.2010.08.013 20832290 PMC3010448

[B49] Ferrante RJ Kowall NW Richardson EP Proliferative and degenerative changes in striatal spiny neurons in Huntington's disease: a combined study using the section-Golgi method and calbindin D28k immunocytochemistry J Neurosci 1991 12 3877 3887 1836019 10.1523/JNEUROSCI.11-12-03877.1991 PMC6575286

[B50] Angeli S Shao J Diamond MI F-actin binding regions on the androgen receptor and huntingtin increase aggregation and alter aggregate characteristics PLoS One 2010 5 2 e9053 10.1371/journal.pone.0009053 20140226 PMC2816219

[B51] Bezprozvanny I Inositol 1,4,5-tripshosphate receptor, calcium signalling and Huntington's disease Subcell Biochem 2007 45 323 335 10.1007/978-1-4020-6191-2_11 18193642

[B52] Henshall TL Tucker B Lumsden AL Nornes S Lardelli MT Selective neuronal requirement for huntingtin in the developing zebrafish Hum Mol Genet 2009 18 24 4830 4842 10.1093/hmg/ddp455 19797250 PMC2778375

[B53] Reiner A Del Mar N Meade CA Yang H Dragatsis I Neurons lacking huntingtin differentially colonize brain and survive in chimeric mice J Neurosci 2001 21 19 7608 7619 11567051 10.1523/JNEUROSCI.21-19-07608.2001 PMC6762912

[B54] Cartier L Galvez S Gajdusek DC Familial clustering of the ataxic form of Creutzfeldt-Jacob disease with Hirano bodies J Neurol Neurosurg Psychiatry 1985 48 3 234 238 10.1136/jnnp.48.3.234 2984334 PMC1028256

[B55] Hirano A Hirano bodies and related neuronal inclusions Neuropath Appl Neurobiol 1994 20 3 11 10.1111/j.1365-2990.1994.tb00951.x 8208338

[B56] Tomanaga M Hirano body in extraocular muscle Acta Neuropathol 1983 60 309 313 10.1007/BF00691883 6310930

[B57] Gibson PH Tomlinson BE Numbers of Hirano bodies in the hippocampus of normal and demented people with Alzheimer's disease J Neurol Sci 1997 33 199 206 903782 10.1016/0022-510x(77)90193-9

[B58] Maselli AG Davis R Furukawa R Fechheimer M Formation of Hirano bodies in *Dictyostelium *and mammalian cells induced by expression of a modified form of an actin-crosslinking protein J Cell Sci 2002 115 (Pt 9):1939 1949 11956325 10.1242/jcs.115.9.1939

[B59] Davis RC Furukawa R Fechheimer M A cell culture model for investigation of Hirano bodies Acta Neuropathol 2008 115 2 205 217 10.1007/s00401-007-0275-9 17978823

